# Leveraging community engaged research partnerships for crisis and emergency risk communication to vulnerable populations in the COVID-19 pandemic

**DOI:** 10.1017/cts.2020.47

**Published:** 2020-05-15

**Authors:** Mark L. Wieland, Gladys B. Asiedu, Kiley Lantz, Adeline Abbenyi, Jane W. Njeru, Ahmed Osman, Miriam Goodson, Yahye Ahmed, Luz E. Molina, Chyke A. Doubeni, Irene G. Sia, for the Rochester Healthy Community Partnership COVID-19 Task Force

**Affiliations:** 1 Division of Community Internal Medicine, Mayo Clinic, Rochester, MN, USA; 2 Kern Center for the Science of Healthcare Delivery, Mayo Clinic, Rochester, MN, USA; 3 Division of Infectious Diseases, Mayo Clinic, Rochester, MN, USA; 4 Center for Healthy Equity and Community Engagement Research, Mayo Clinic, Rochester, MN, USA; 5 Intercultural Mutual Assistance Association, Rochester, MN, USA; 6 Alliance of Chicanos, Hispanics, and Latin Americans, Rochester, MN, USA; 7 Somali American Social Service Organization, Rochester, MN, USA; 8 Department of Family Medicine, Mayo Clinic, Rochester, MN, USA

**Keywords:** COVID-19, community-engaged research, immigrant health

## Abstract

Community engagement is important for reaching vulnerable populations in the coronavirus disease 2019 (COVID-19) pandemic. A risk communication framework was implemented by a community-engaged research (CEnR) partnership in Southeast Minnesota to address COVID-19 prevention, testing, and socioeconomic impacts. Bidirectional communication between Communication Leaders and community members within their social networks was used by the partnership to refine messages, leverage resources, and advise policy makers. Over 14 days, messages were delivered by 24 Communication Leaders in 6 languages across 9 electronic platforms to 9882 individuals within their networks. CEnR partnerships may effectively implement crisis and emergency risk communication to vulnerable populations in a pandemic.

## Introduction

Crisis and emergency risk communication frameworks are currently being applied in the public health response to the coronavirus disease 2019 (COVID-19) pandemic to encourage public participation in disease prevention and containment. Common principles of these frameworks are to be correct, credible, and respectful, to promote action and to engage with communities in order to empower decision-making [1,2].

Effective application of risk communication frameworks depends, in part, on reaching vulnerable populations with a history of social injustice, health disparities, and limited access to health information. Racial minorities have been disproportionately impacted by COVID-19. For example, the age-adjusted death rate in New York City for blacks and Hispanics as of April 6, 2020, was approximately double the rate among whites [3]. Thus, there is an urgent need for effective channels of risk communication with vulnerable populations [4].

Vulnerable populations and minorities are more likely to have communication gaps due to socioeconomic disadvantage, low health literacy, immigration status, and limited English proficiency [5], compounded by language and cultural discordance and mistrust of health institutions [6]. The Centers for Disease Control and Prevention (CDC) Crisis and Emergency Risk Communication Manual describes three levels of community engagement (low, medium, high) and acknowledges that a high level of engagement that starts prior to any emergency is needed to reach vulnerable populations in times of crisis [1].

Community-engaged research (CEnR) partnerships are uniquely positioned to operationalize pandemic risk communication frameworks among vulnerable populations. CEnR partnerships, characterized by collaboration between community members and researchers through all phases of research, are increasingly ubiquitous in the United States across disciplines, population groups, and geography [7]. These partnerships have access to large networks of vulnerable groups through their focus on health equity, and community partners have organizational and technical capacity for interfacing with these populations in a research and evaluation context [8]. CEnR partnerships have already laid the foundation for engagement through prior work, thereby addressing the risk communication principles of credibility, respect, and relationships that pre-date a crisis while empowering community decision-making. Likewise, CEnR partnerships empower community decision-making, which is a critical risk communication process [1].

This study aims to demonstrate the use of a CEnR health partnership with vulnerable populations leveraging its social networks, credibility, and technical expertise to promote bidirectional crisis and emergency risk communication for the COVID-19 pandemic.

## Methods

Community and academic partners from Rochester Healthy Community Partnership (RHCP), a CEnR partnership with a 15-year history of participatory research with immigrant populations in Southeast Minnesota [9], adopted the CDC Crisis and Emergency Risk Communication framework for co-creation of an intervention framework aimed at populations with limited English proficiency. RHCP community partners observed that credible COVID-19 information was being produced, but it was not reaching immigrant communities.

### Intervention Development

COVID-19 message maps were developed by RHCP community and academic partners. Message maps are a framework used to create compelling messages for specific audiences; each concise message is supported by 2–3 facts [10]. Message content was consistent with communication from regional and national public health officials and Mayo Clinic and focused on three constructs: COVID-19 prevention and containment; SARS Coronavirus-2 testing; and social and economic impacts of COVID-19 [11]. As COVID-19 facts changed, the same process was used to generate biweekly “reports” throughout the intervention that included new messages or refinements of previous messages. Materials were professionally translated into six languages by an institutional community partner.

### Recruitment and Training of Communication Leaders

Communication Leaders were recruited by RHCP community partners to deliver messages based on their credibility and trustworthiness within subset communities. The majority of Leaders (22/24) had worked with RHCP in previous projects. A single virtual meeting was held with Communication Leaders and RHCP partners to review the intervention framework and messages.

### Intervention

**Fig. 1.**
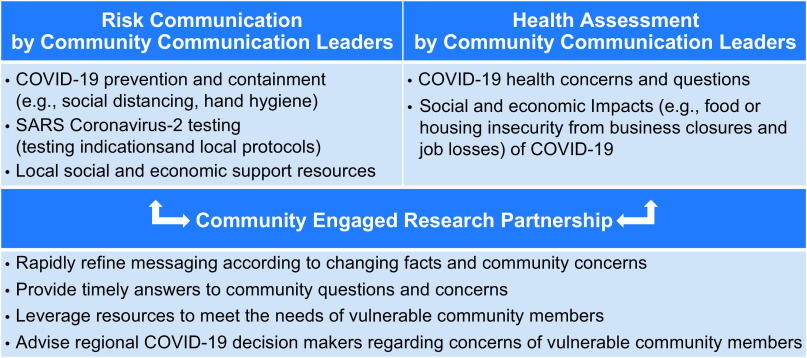
Intervention framework.

COVID-19 messages were delivered by 24 bilingual Communication Leaders within their social networks. Because of physical distancing, messages were mostly delivered electronically. There were no guidelines around communication mediums (e.g., voice calls, text messaging, social media) or mechanisms (e.g., video, text, audio). For example, some Communication Leaders sent the messages in full, others broke them up into smaller message components, and others used a device to record a video of themselves communicating the key components of each message. Recipients were encouraged to amplify the messages to their social networks. Additionally, Communication Leaders solicited community health and socioeconomic concerns through the same platforms. This bidirectional communication with vulnerable community members was used by RHCP to enhance subsequent messaging, leverage resources to meet community needs, and advise regional decision makers (Fig. 1).

Communication Leaders and RHCP partners met daily via an hour-long teleconference for three purposes. First, Communication Leaders shared their progress for all to learn emerging best practices from others. Second, message refinement and generation of new messages were achieved in real time in response to community feedback as well as rapidly changing COVID-19 facts and resources. Third, questions were answered in real time by infectious disease experts (academic partners) or community resource experts (community partners). In-between phone calls, Communication Leaders and RHCP partners communicated by text messages and email.

Examples of new messages or message modifications based on community feedback included specific COVID-19 “myth-busters,” advice for funeral practices, and advice for physical distancing while outdoors. Examples of bidirectional communication that informed community resource allocation included targeted distribution of food and face coverings to vulnerable community members identified by Communication Leaders. Finally, feedback from community members was used to inform regional decision makers regarding COVID-19 testing procedures so that messaging was consistent regardless of where the individual received their usual healthcare.

### Evaluation

Rapid evaluation and assessment methods [12] were used for participatory evaluation of the intervention, which informed continuous intervention refinement. The evaluation interval was the first 14 days of intervention implementation: March 27 to April 10, 2020. We assessed intervention reach, acceptability, and feasibility. We also used qualitative data to map findings to the CDC Crisis and Emergency Risk Communication framework.

Sources of data for evaluation were (1) collated tracking sheets from Communication Leaders documenting daily outreach numbers and communication mechanisms, (2) summary notes from daily teleconferences with Communication Leaders, and (3) summaries of semi-structured interviews with Communication Leaders who provided status updates on what strategies were working, how they had adapted the intervention, and suggestions for improvement. Acceptability and feasibility of the intervention were assessed by the interviews with Communication Leaders and by summary notes from daily teleconferences.

Tracking data were reported as frequencies to assess intervention reach. Content analysis conducted on notes from teleconferences and interviews was entered in NVivo 12 software (QSR International, Pty. Ltd.). Codes were assigned to note text representing concepts in the data to facilitate queries for analysis. Analysis was completed across subgroups and mapped to the CDC Crisis and Emergency Risk Communication framework.

## Results

The intervention was delivered by 24 Communication Leaders (11 Somali, 6 Hispanic, 2 Cambodian, 3 South Sudanese, 1 Anuak, 1 Ethiopian). In addition to the three main message maps, eight COVID-19 updates were translated and disseminated. In addition to bidirectional communication between all partners on daily telephone conferences, 40 emails, text messages, or phone calls were sent to Communication Leaders in response to new COVID-19 developments or community concerns.

### Intervention Reach

A total of 9882 individuals received messages from Communication Leaders through 9 different communication platforms over a 14-day interval (Table 1). The most common communication platforms were Facebook, voice telephone calls, and text messaging (conventional, WhatsApp, and Viber).

**Table 1.** Number of individuals to whom COVID-19 messages were distributed and communication mediums used

Communication medium (no.)	Ethnic group						Total (medium)
	Anuak	Cambodian	Ethiopian	Hispanic	Somali	South Sudanese	
Email				48	7		**55**
Facebook	80	3847	240	1940	750	300	**7157**
Facebook Messenger				22			**22**
In-Person				40	249		**289**
Instagram				9			**9**
Telephone call	15		3		1283	1	**1302**
Text message				33	75	57	**165**
WhatsApp		45		54	670		**769**
Viber			114				**114**
**Total (ethnic group)**	**95**	**3892**	**357**	**2146**	**2304**	**358**	**9882**

### Acceptability and Feasibility

All of the Communication Leaders judged the intervention to be highly relevant and responsive to community needs in a time of crisis. This led to a perception of empowerment for Communication Leaders and their communities in facing the pandemic. Feasibility of applying the framework during the initial 14-day interval was demonstrated to be high in the context of very motivated community partners. Some Communication Leaders reflected that the logistics of applying the intervention framework would have to be flexible in order to sustain momentum in the future. For example, as the economy begins to re-open, meeting times and frequency may have to change to accommodate Communication Leaders’ increasingly busy lives.

### Risk Communication Framework

Table 2 summarizes evaluation findings mapped to the CDC Crisis and Emergency Risk Communication phases of preparation, initial messaging, maintenance, and resolution. In the preparation phase, credibility of RHCP as a partnership and preexisting trust by vulnerable communities was identified as an important intervention facilitator. In the initial implementation phase, action-oriented messages were generated in a participatory way with RHCP partners. This process was perceived as adding credibility to the messages by fully incorporating community voice while staying true to the facts. In the maintenance phase of implementation, daily bidirectional communication was important to revise message content, generate new messages in response to community concerns, and connect community groups to existing resources. RHCP served as a source of strength for Communication Leaders, which enabled community ownership of the intervention.

**Table 2.** Strategies for implementation of centers for disease control and prevention communication and emergency risk communication phases with vulnerable populations in the COVID-19 pandemic by a community engaged research partnership

Communication and emergency risk communication phases	Implementation strategies by community engaged research partnership
**Preparation**	
Develop partnership	Intervention was built by an established CEnR partnership with preexisting trust and credibility within regional immigrant communities. Shared ownership of the COVID-19 risk communication project was agreed upon by community and academic task force partners.^*^
Draft and test messages	COVID-19 message maps with content from credible sources were co-created by community and academic partners. Additional biweekly “reports” and videos were developed in partnership with community leaders for distribution through several electronic mediums (Table 1). Messages were tailored to meet each language group’s needs. Beyond the main messages (Fig. 1), additional messages included situational updates and responses to frequently asked questions solicited by Communication Leaders.
Create plan	Dissemination plan was discussed at an initial teleconference between Communication Leaders and task force members. The plan was augmented daily via teleconferences with the task force.
Determine approval process	Equal decision-making on approval process of all messages by task force members. Messages were reviewed and edited by Communication Leaders from each language group before translation. Academic partners reviewing message content included an infectious diseases specialist, and messages were cross-referenced daily with CDC and regional and local health department, as well as WHO website content.
**Initial**	
Explain risk	Messages targeted ways to stop the spread and transmission of COVID-19. Task force subgroups focused on reaching high risk elderly populations and those with chronic conditions, while others focused on reaching youth who often expressed sentiments of lower personal risk.
Promote action	All messages were action-oriented with specific steps to take for COVID-19 prevention, testing, and associated socioeconomic stressors. Messages were reenforced regularly and altered in response to community questions and stated actions.
Describe response efforts (organization’s credibility)	The task force maintained daily bidirectional contact with Communication Leaders. In addition to teleconferences, this included individual phone calls and text messages between task force members. A participatory approach promoted ownership of the intervention by Communication Leaders and their community partners. Credibility of the CEnR partnership was perceived as critical to the intervention success.
**Maintenance**	
Provide background information	Initial messaging included background information about and testing for COVID-19. These basic messages were reenforced throughout the maintenance phase.
Explain ongoing risks	Concerns and questions brought forward by community leaders from their communities revealed the magnitude of the crisis and how communities were assessing personal risk. Most of these questions, including those about changing perceived risk, could be answered by task force partners in real time during daily teleconferences in a culturally focused manner (e.g., how to address culture-specific mourning and burial of the deceased). If not, then community referrals were initiated through existing partnership networks.
Segment audiences	Messages were adapted for each language group for cultural context. Messages were also adapted for youth audiences by a task force subgroup. Finally, messages were adapted for families and contacts of those who tested positive for COVID-19.
Address rumors	Rumors were directly addressed in real time during daily teleconferences with Communication Leaders, who curated these rumors from members of their social networks. Rumors commonly centered on home remedies for COVID-19, treatment efficacy, vaccine availability, and risk misperception.
**Resolution**	
Motivate vigilance	Complacency was discouraged through sustainability of bidirectional, regular task force communications and through continuous application of community engagement principles that place equal ownership of the process with community and academic partners.
Discuss and document lessons learned	Lessons learned were catalogued from two sources: notes from daily task force teleconferences and weekly semi structured interviews with Communication Leaders. Through rapid analysis of these data sources, messages were refined, community resources were leveraged, and concerns of immigrant communities were expressed to regional COVID-19 decision makers.
Evaluate and revise plans	Rapid evaluation was conducted in real time as above. Examples of revised plans that arose from these evaluations included more coordinated messaging around social and economic resources in the community, a more streamlined COVID-19 testing protocol for individuals with limited English proficiency (adapted by healthcare institution), more solution-based messages, more message repetition, and new messages of empathy and hope.

Abbreviations: COVID-19, coronavirus disease 2019; CEnR, community engaged research; CDC, Centers for Disease Control and Prevention; WHO, World Health Organization.

* Task force members included community and academic leaders, Communication Leaders, other volunteers and representatives from regional community-based organizations.

## Discussion

This study describes the ways an existing CEnR partnership leveraged its credibility and trust with vulnerable populations for risk communication in the COVID-19 pandemic. By adopting a risk communication framework, co-creating messages with community leaders and health experts, and modifying messages daily, Communication Leaders felt supported in disseminating accurate COVID-19 messages to their networks. Rapid evaluation and assessment methods through tracking, daily teleconferences, and weekly interviews allowed the team to adapt the messages and connect community members to resources in real time. Community concerns were used to influence local testing policies and practices by healthcare partners. In these ways, the intervention is one example of how previously articulated best practices for pandemic risk communication to vulnerable populations may be applied [13].

CEnR partnerships have grown rapidly in recent years within the health equity space [7]. This study demonstrated that CEnR partnerships are uniquely poised to respond to pandemic risk communication needs with at-risk communities through ready access to disease content expertise from academic partners and community expertise from community partners. Furthermore, this study described the ways in which a CEnR partnership leveraged community capacity for rapid evaluation and data collection through past research experiences. Processes and products from this intervention may be adapted by other CEnR partnerships for local contexts. For example, two additional CEnR partnerships within the Mayo Clinic enterprise are in the early stages of applying components of the intervention framework in Minnesota and Florida. Finally, implementation of the RHCP intervention framework has continued into its second month with engaged Communication Leaders and community partners, which provides preliminary evidence for the sustainability of the intervention.

The study has limitations. We did not assess dissemination of the messages beyond the initial distribution from Communication Leaders. Therefore, the full intervention reach cannot be assessed across social networks (message amplification). The number of individuals reached by Communication Leaders may have been overestimated if individuals received messages from more than one Communication Leader via overlapping networks. Engagement data from social media platforms were not collected. Furthermore, risk-related behaviors and outcomes were not assessed. Finally, CEnR partnership work is highly contextual, so this process may not be generalizable to some partnerships.

## Conclusion

By leveraging existing networks and credibility, CEnR partnerships may effectively implement crisis and emergency risk communication to vulnerable populations in the COVID-19 pandemic.
